# Subacute voluntary care provision, what about Yalom?

**DOI:** 10.1177/10398562231158918

**Published:** 2023-02-23

**Authors:** Klaus Martin Beckmann, Brendan Pepe

**Affiliations:** 5723 Meadowbrook, QLD; 95793 Mansfield, QLD

Dear Sir,

The prevalence of mental health morbidity is on the increase, particularly in the wake of the COVID-19 pandemic. With an ever-increasing demand for resources, remaining gaps in service provision, inpatient units and community mental health services have seen an expansion into other areas, such as subacute care services (SCS).

We are writing to highlight SCS in Australia, which increasingly feature in RANZCP journals. The 2022 Sydney RANZCP AGM included presentations showcasing SCS.^1^ On review of Australian literature on SCS and their policies, guidelines and model of service delivery, we do not find mention of group therapeutic factors (GTFs) yet believe these are an important integral part to SCS.

Both Victoria and Queensland boast SCS. In Victoria, PARCS (Prevention and Recovery Care Service) opened early this century, and in Queensland, SCS opened Step-Up-Step-Down Service (SUSDS) late last decade. SCS provide community-based, recovery-orientated residential care. Staffing typically is provided by public health service and non-government organisation (NGO) personnel. Early evidence, based on mental health outcomes, suggests that patients benefit from SCS admission.^2^ Overall admissions are voluntary. SCS multidisciplinary teams offer diverse bio-psycho-social therapies individually and as groups in a recovery and life skills model. Moreover, therapeutic interactions occur out-of-session in the interaction amongst clients themselves, friends and relatives, staff from NGO care providers, and public mental health staff. We believe this variety of opportunities is an integral part to treatments at SCS. Evidence published developing a rationale as to why some patients can do well at SCS to this date does not include GTF. We find that this is an oversight. What happens out-of-sessions cannot be underestimated.

Yalom’s^3^ 12 group therapeutic factors found validity in contexts other than group therapies.^4^ The 12 factors are instillation of hope, universality, imparting of information, altruism, simulation of the primary family, development of social skills, imitative behaviours, interpersonal learning input and output, group cohesiveness, catharsis and existential factors.

We believe that GTF has so far been overlooked; group dynamics contribute to the therapeutic momentum at SCS. We believe raising awareness and being mindful and skilful about addressing GTF may well enhance what SCS can do to support patient’s recovery. Outcome research, audits and appraisal of SCS should consider GTF.
